# Prevalence of methicillin resistant *Staphylococcus aureus*, multidrug resistant and extended spectrum β-lactamase producing gram negative bacilli causing wound infections at a tertiary care hospital of Nepal

**DOI:** 10.1186/s13756-018-0408-z

**Published:** 2018-10-08

**Authors:** Narbada Upreti, Binod Rayamajhee, Samendra P. Sherchan, Mahesh Kumar Choudhari, Megha Raj Banjara

**Affiliations:** 1 Central Department of Microbiology, Tribhuvan University, Kirtipur, Nepal; 2 National College (Tribhuvan University), Khusibu, Kathmandu, Nepal; 3 Department of Infectious Diseases and Immunology, Kathmandu Research Institute for Biological Sciences (KRIBS), Lalitpur, Nepal; 4 Department of Global Environmental Health Sciences, School of Public Health and Tropical Medicine, Tulane University, New Orleans, LA USA; 5 KIST Medical College and Teaching Hospital, Imadole, Lalitpur, Nepal

**Keywords:** Wound infection, Methicillin resistant *Staphylococcus aureus*, ESBL, Multidrug resistant, Nepal

## Abstract

**Background:**

Treatment and prevention of wound infection continues to be a challenging issue in clinical settings of Nepal especially in the context of globally growing problem of antimicrobial resistance. Study on opportunistic pathogens and sensitivity to commonly prescribed local antimicrobial agents are cardinal to reduce the disease burden of wound infections. The aim of this study was to determine the prevalence and antimicrobial susceptibility pattern of methicillin resistant *Staphylococcus aureus* (MRSA) and extended spectrum β-lactamase (ESBL) producing bacteria from wound infections of patients at a tertiary care hospital in Nepal.

**Methods:**

Pus specimens were processed using standard microbiological procedures. Antimicrobial susceptibility test was performed following the modified Kirby Bauer disc diffusion technique. Clinical information of patients was obtained from preformed questionnaire and hospital record.

**Results:**

One hundred eighty two pus specimens from wounds of different body parts: leg, hand, backside, abdominal part, foot, breast and chest, head and neck region were collected and analyzed; 113 bacterial isolates were isolated showing the overall bacterial growth rate of 62%, where the highest rate was among patients of ≤10 years age group (82.1%). A higher rate (68.5%) of bacterial isolates were from inpatients (*p* < 0.05). Among 116 bacterial isolates, *Staphylococcus aureus* was the most predominant bacteria (56.9%) followed by *Escherichia coli* (8.6%), coagulase negative staphylococci (7.8%), *Acinetobacter* spp. (5.2%), *Klebsiella pneumoniae* (5.2%), *Pseudomonas aeruginosa* (4.3%), *Enterococcus* spp. (4.3%), *Citrobacter freundii* (2.6%), *Proteus vulgaris* (1.6%) and *P. mirabilis* (0.9%). Both Gram positive (73.3%) and negative (78.8%) isolates showed high frequency of sensitive to gentamycin.

**Conclusion:**

Among *S. aureus* isolates*,* 60.6% were MRSA strains, whereas 40% of *K. pneumoniae* and 33.3% of *C. freundii* were ESBL producing bacteria followed by *E. coli* (25%). It is thus paramount to address the burden of silently and speedily increasing infections caused by drug resistant strains of MRSA and ESBL in Nepal.

**Electronic supplementary material:**

The online version of this article (10.1186/s13756-018-0408-z) contains supplementary material, which is available to authorized users.

## Background

Wound infections result after the active interactions that takes place between a host, a potential pathogen and the surrounding extrinsic factors. The intensity of wound infections may range from a simple self-healing to a severe and life threatening [1]. Tissue invasion by bacterial pathogens is determined by the location of wound [2]. The common bacterial pathogens isolated from wound infections are *Staphylococcus aureus*, *S. epidermidis*, *S. pyogenes,* coagulase negative staphylococci (CoNS), *Acinetobacter* spp., *Pseudomonas* spp., *Escherichia coli*, *Klebsiella* spp., *Proteus* spp., *Enterobacter* spp., *Citrobacter* spp., and anaerobes such as *Clostridium* spp. and *Peptostreptococcus* spp. [3, 4]. Acquisition of drug resistance by these pathogenic strains has posed serious challenges for the remedy and management of wound infections around the world [5]. Wound infections can be monomicrobial or polymicrobial [6]. The presence of bacterial pathogens in wound infections is not uncommon but all wounds do not support the same range and number of species [7]. Hospital-acquired wound infections are the leading cause of morbidity hence, proper management of wound infection in clinical settings is paramount [8]. The treatment of wound infections is being more challenging due to methicillin resistant *S. aureus* (MRSA), involvement of polymicrobial flora and fungi [9]. In addition, antimicrobial resistance (AMR) is creating a serious problem in all clinical settings and AMR has become the biggest public health threat globally [10].

MRSA, a leading strain of wound infections, involves significant areas of skin or deeper soft tissues like abscesses, cellulitis, burns or infected deep ulcers [11]. Extended spectrum β- lactamase (ESBL) producing Enterobacteriaceae are also in frontline of wound infections. In ESBL, positive strains plasmid mediated AmpC enzymes, and carbapenem hydrolyzing β- lactamase (carbapenemases) conferred resistance to the newer β- lactam antimicrobials [12]. ESBL have been reported most frequently in *Escherichia coli* and *Klebsiella* spp. including other bacterial species such as *Salmonella enterica*, *P. aeruginosa*, and *Serratia marcescens* [13]. This surge in antimicrobial resistance further delays wound healing and the infection becomes more worst which increases hospital stay, prolongs trauma care, and high medical costs [14]. On the other hand, most of the clinical laboratories in underdeveloped countries are not equipped with testing facilities to detect ESBL producing bacteria. In Nepal, there is scanty data on the prevalence of ESBL-producing bacteria causing wound infections. The goal of this study was to determine the prevalence of MRSA, multidrug resistant and ESBL producing Gram negative bacilli from wound infections of patients visiting KIST Medical College and Teaching Hospital, Lalitpur, Nepal. Early reporting of drug resistant pathogens and evidence-based treatment algorithm can control the wound infections.

## Methods

### Study site and population

A descriptive cross-sectional study was designed and carried out to determine the bacteriological profile of wound infections. MRSA, MDR and ESBL producing bacteria were identified from the pus samples of patients with wound infection visiting KIST Medical College and Teaching Hospital, Kathmandu, Nepal from November 2014 to August 2015. A total of 182 pus and Fine Needle Aspirate specimens were collected from patients with clinical features of wound infection like patients with pain, complaints of regular discharge, foul smelling and red swelling. During the study, patients of all age groups and both genders from out-patients (39/182) and in-patients (143/182) were included. Patients who were admitted in the hospital for more than 3 days and/or in prior antibiotic treatment and anaerobic wound infections were excluded from this study.

### Sampling procedure

Pus specimens were collected from elective surgery wounds of hospital wards [surgical, post- operative, trauma, orthopedic, ENT (eye-nose-throat), gynecology wards], open and dressed wounds. Sterile cotton swabs and fine needle syringes (FNS) were used to collect pus samples from open wounds then each sample was labeled properly with date/time of sample collection, collection method and the patient’s details. Swabs from open wounds were aseptically collected after cleaned off while pus from dressed wounds were collected after removing the dressing items. The information of each patient was recorded such as site of infection, signs and symptoms, other underlying diseases, and prior antibiotics administration. Before collecting the sample, the area was rinsed with sterile normal saline and then a sterile cotton swab was gently rolled over the surface of the wound. The swab with pus was kept in a sterile test tube with cap where details was labeled properly. For the collection of pus sample from deep wounds, FNS was used. Specimens were collected from wounds of different body parts: leg, hand, back part of body, abdominal part, foot region, breast and chest part, head and neck region. Amies transport medium was used to transport the collected specimens. For Fine Needle Aspiration Cytology (FNAC), the syringe was properly capped, labeled and dispatched to the laboratory immediately.

### Processing of samples

#### Macroscopic examination of samples

Among 182 pus specimens collected, 56 (30.8%) were from the leg region, 43 (23.6%) from hand, 15 (8.2%) from back part of body, 14 (7.8%) from abdominal part, 15 (8.2%) from foot region, 6 (3.3%) from breast and chest part, and 33 (18.1%) were from head and neck region wounds. All the specimens were visually examined for consistency, color, turbidity, presence or absence of blood depending upon the type and site of wound. Additionally, pus swabs were observed whether they were labeled correctly or not.

#### Microscopic examination of samples

After transportation of specimens to the laboratory, Gram staining of each specimens was performed [15].

### Culture of specimens and identification of isolated bacteria

Pus specimens were inoculated into Chocolate agar, Blood agar, MacConkey agar, Nutrient agar and Potato Dextrose agar plates as per the clinical laboratory guidelines [16]. The preliminary identification of the isolated bacteria was done based on colony form, size, shape, pigmentation, margin, and elevation. The isolated organisms were identified by performing different biochemical tests and Gram staining then antimicrobial susceptibility tests were performed. In case of no growth after 24 h of incubation further incubation was done up to 48 h at 37 °C. After proper incubation period, the culture plates were examined for microbial growth. In every case, each plate was carefully observed. Then, biochemical tests were performed in sterile media for the identification of bacterial isolates. Identification of Staphylococci spp. was done by Gram staining, catalase test, slide coagulase and tube coagulase test. Similarly, Gram negative strains were identified based on result of different biochemical tests; Oxidase, Catalase, Methyl Red (MR), Voges Proskauer (VP), Citrate utilization, Urea Hydrolysis, Triple Sugar Iron agar (TSI), Sulfide Motility and Indole test. Colony morphology and microscopic observation were taken in account for identification of *Candida* spp.

### Examination of antimicrobial susceptibility pattern of isolated organism

Antimicrobial susceptibility pattern was performed for isolated and identified bacteria from pus samples following the modified Kirby Bauer disc diffusion technique. A dilution of the identified organism was prepared comparing with the standard 0.5 McFarland turbidity which was used to swab over the Mueller Hinton agar (MHA) medium for the antimicrobial susceptibility test (AST). Discs of antibiotic used for Gram positive bacteria were ampicillin (10 μg), cefotaxime (30 μg), gentamycin (10 μg), ciprofloxacin (5 μg), trimethoprim + sulfamethoxazole (25 μg), cefoxitin (30 μg), amikacin (30 μg) and tetracycline (30 μg) whereas antibiotics used for Gram negative organisms were ampicillin (10 μg), trimethoprim + sulfamethoxazole (25 μg), gentamycin (10 μg), ciprofloxacin (5 μg), cefazolin (30 μg), ceftriaxone (30 μg), cefotaxime (30 μg), amikacin (30 μg), piperacillin (100 μg), tobramycin (10 μg), imipenem (10 μg), and meropenem (10 μg). After 24 h of incubation period at 37 °C, the zone of inhibition (ZOI) was measured then the results were analyzed according to the guidelines issued by the Clinical Laboratory Standard Institute (CLSI - M100-S25, 2015) [16]. Isolates resistant to two or more antimicrobial classes were reported as multi drug resistant (MDR) strains. Antimicrobials and their doses were selected based on prescription frequency by physician and availability in the study setting. Minimum inhibitory and bactericidal concentration (MIC and MBC) of used antimicrobials were not determined due to unavailability of all antimicrobials powder at the time of study period.

### Screening and confirmation for ESBL producers

Enterobacteriaceae isolates were screened for possible ESBL producing bacteria using antibiotic discs of cefotaxime (30 μg), ceftazidime (30 μg), ceftriaxone (30 μg) and aztreonam (30 μg) [17]. According to the guidelines, bacterial isolates showing ceftazidime < 22 mm, and cefotaxime < 27 mm are the possible ESBL producer. The suspected ESBL producer strains were subjected to double disc synergy test (DDST) for the confirmation of ESBL producing Enterobacteriaceae [18].

### Statistical analysis

All data were examined using iBM SPSS version 21.0. Frequencies were calculated for categorical variables. Chi-square test was calculated to analyze significant difference at 95% of confidence level, *p* value of < 0.05 was considered significant, unless otherwise noted.

### Quality control

All prepared biochemical and streaking media were checked for their sterility. Strains of *E. coli* ATCC 25922 and *S. aureus* ATCC 25923 were used as reference strains for quality control of AST and biochemical tests. The same strain of *E. coli* was also considered as a negative control during the screening and phenotypic confirmation (DDST) tests of ESBL producing Gram-negative bacilli.

## Results

### Bacterial growth

**Fig. 1:**
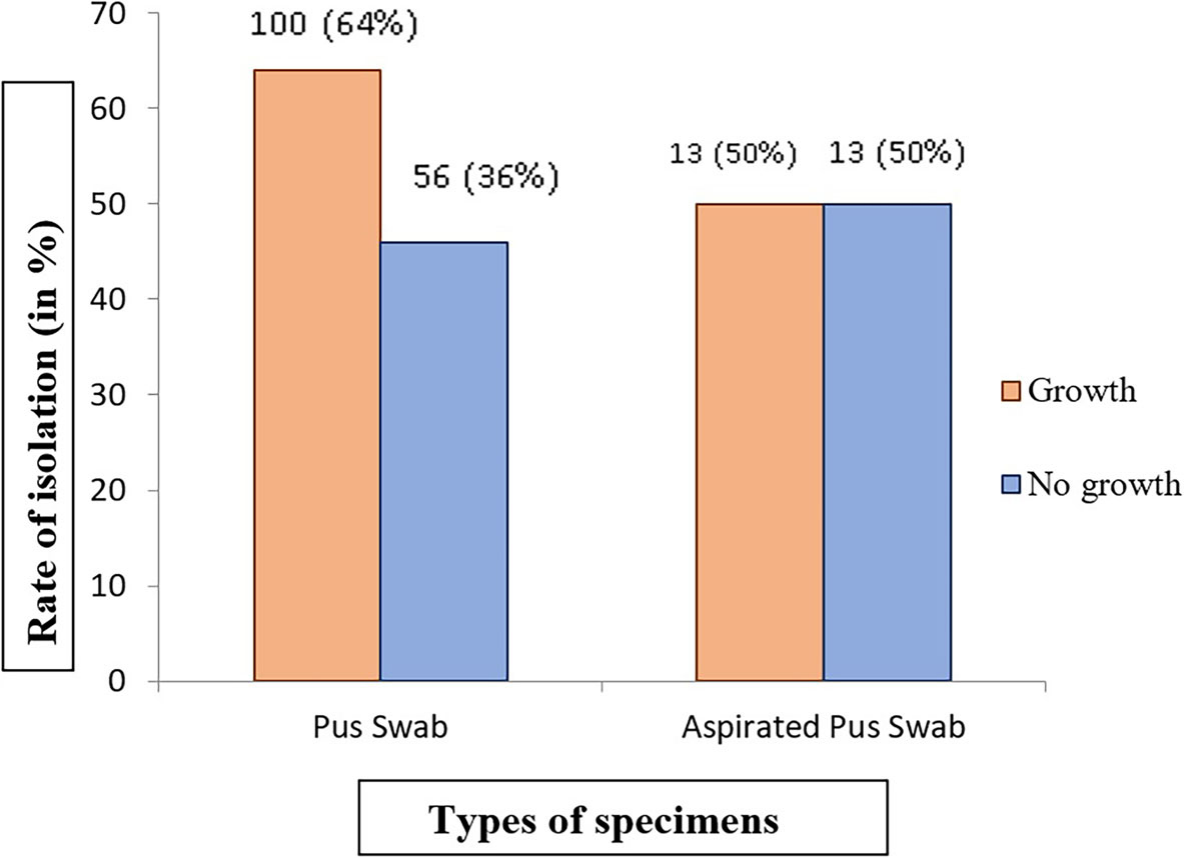
Percentage of bacterial growth in pus swab and aspirated pus swab

A total of 182 samples were collected and examined from hospital patients with clinical features of wound infection, 113 (62%) specimens were positive for aerobic bacterial growth. Out of 116 bacterial isolates obtained from 113 positive samples, 83 (71.6%) bacterial isolates were Gram positive and 33 (28.4%) isolates were Gram negative. Among processed specimens, 64% (100/156) of pus swabs and 50% (13/26) of aspirated pus specimens have shown aerobic bacterial growth (Fig. 1). Out of 113 specimens positive for aerobic bacterial culture, polymicrobial growth was observed in 3 (2.7%) specimens where combinations of *S. aureus* - *Acinetobacter* spp., *S*. *aureus* - *Citrobacter freundii* and *Enterococcus* spp. - *Candida* spp. were reported. High incidence of MRSA 60.6% (40/66), MDR (80% of *E. coli*, 68.2% of *S. aureus*, 80% of *P. aeruginosa*, 77.7% of CoNS and 50% of *Proteus* spp.) and ESBL (25% of *E. coli*, 40% of *K. pneumoniae*, and 33.3% of *C. freundii*) producing isolates were reported in this study.

Sixty two (34.1%) specimens processed were collected from the leg, 36 (19.8%) from hand, 16 (8.8%) from backside, 15 (8.2%) from abdominal, 22 (12.1%) from foot, 13 (7.1%) from breast and chest, 18 (9.9%) from head and neck part. Majority of patients (86%) were presented with fever, lethargy and muscle pain at the time of sample collection. None of the patients were reported with any underlying diseases. Patients who had other infections and antibiotic treatment were excluded from the study subject.

### Wound infection in relation with demographic characteristics of the patients

Eighty one (44.5%) samples were from male patients and among them 45 (55.5%) samples showed aerobic bacterial growth, while 101 (55.5%) samples were from female patients, and 68 (68.3%) samples were positive for aerobic bacterial growth but there was no significant difference in between aerobic bacterial growth and gender of patients (*p* > 0.05) (Table 1). Highest rate of wound infection was observed among patients of age group ≤10 years (82.1%), followed by patients of age group 71–80 years (77.8%).

**Table 1:** Socio-demographic features of the patients and ratio of wound infection

Demographic features	Infected [No. (%)]	Not infected [No. (%)]	Total [No. (%)]
Sex			
Male	45 (55.6)	36 (44.4)	81 (44.5)
Female	68 (67.3)	33 (32.7)	101 (55.5)
Total	113 (62.1)	69 (37.9)	182 (100)
Age in years			
≤ 10	23 (82.1)	5 (17.9)	28 (15.4)
11–20	18 (60.0)	12 (40.0)	30 (16.5)
21–30	12 (44.4)	15 (55.6)	27 (14.9)
31–40	21 (65.6)	11 (34.4)	32 (17.6)
41–50	9 (40.9)	13 (59.1)	22 (12.0)
51–60	15 (68.2)	7 (31.8)	22 (12.0)
61–70	8 (66.7)	4 (33.3)	12 (6.6)
71–80	7 (77.8)	2 (22.2)	9 (5.0)
Total	113 (62.00)	69 (38.00)	182 (100)

### Growth pattern in outpatient and inpatient departments

One hundred forty three samples were from inpatient department (from different wards) and 39 samples were from outpatient department. Out of 143 samples from inpatient, 98 (68.5%) were positive and out of 39 samples from outpatient, 15 (38.5%) were positive for bacterial growth. Type of patients based on department had a positive correlation with aerobic bacterial growth (*p* < 0.05).

Pus specimens were collected from inpatient departments/wards (such as surgical wards, post- operative wards, orthopedic ward, ENT (eye-nose-throat), gynecology wards) and from outpatient department. Eighty nine (48.9%) specimens were from traumatic cases, followed by 57 (31.3%) specimens which were from postoperative cases. The most common bacterial isolate was *S*. *aureus* followed by *E*. *coli.* Out of 116 microbial isolates, 83 (71.6%) were Gram-positive and among them, *S*. *aureus* 66 (79.6%) was the most common isolate followed by CoNS 9 (10.8%), *Enterococcus* spp. 5 (6%) and *Candida* spp. 3 (3.6%). On the other hand, 33 (28.4%) were Gram-negative of which *E*. *coli* 10 (30.3%) was predominant isolate followed by *K*. *pneumoniae* 6 (18.2%), *Acinetobacter* spp. 6 (18.2%), *P*. *aeruginosa* 5 (15.1%), *C*. *freundii* 3 (9.1%), *P*. *vulgaris* 2 (6.1%) and *P*. *mirabilis* 1 (3%). In pus swab, *S*. *aureus* (58%) was the predominant isolate followed by *E*. *coli* (10%) and CoNS (9%). Similarly, in case of aspirates pus samples, *S*. *aureus* (50%) was the highest followed by *K*. *pneumoniae* (18.7%) (Table 2 and Additional file 1).

**Table 2:** Pattern of microbial isolates in wound samples

Type of organism	Type of Specimens				Total	
	Pus swab		Aspirated pus			
	No.	%	No.	%	No.	%
*S*. *aureus*	58	58	8	50	66	56.9
*E*. *coli*	10	10	–	–	10	8.6
*P*. *aeruginosa*	5	5	–	–	5	4.3
CoNS	9	9	–	–	9	7.8
*Acinetobacter* spp.	6	6	–	–	6	5.2
*Enterococcus* spp.	3	3	2	12.5	5	4.3
*C*. *freundii*	1	1	2	12.5	3	2.6
*K*. *pneumoniae*	3	3	3	18.7	6	5.2
*P*. *vulgaris*	2	2	–	–	2	1.6
*P*. *mirabilis*	1	1	–	–	1	0.9
*Candida* spp.	2	2	1	6.3	3	2.6
Total	100	100	16	100	116	100

#### Antibiogram result of gram negative bacteria isolated from patients at KIST Hospital, November 2014 to august 2015

A total of 10 *E*. *coli* were isolated from wound specimens and 80% (8/10) of isolates were sensitive to gentamicin, 60% were sensitive to ciprofloxacin, 50% were sensitive to cefotaxime and 40% were sensitive to cotrimoxazole. All isolates of *E. coli* (100%) were resistant to ampicillin followed by cefazolin (80%) and ceftriaxone (70%). All the isolates of *P*. *aeruginosa* (100%) were susceptible to amikacin, tobramycin and imipenem while 80% of the *P*. *aeruginosa* isolates were sensitive to ciprofloxacin. In contrast, 40% and 60% of *P*. *aeruginosa* isolates were resistant to ceftazidime and piperacillin respectively. Similarly, 83.3% (6/5) of *K*. *pneumoniae* were sensitive to meropenem while 66.7% of isolates were susceptible to ciprofloxacin, gentamycin and amikacin. A total of 50% of the *K*. *pneumoniae* isolates were sensitive to cotrimoxazole and ceftriaxone. All the isolates (100%) of both *Proteus vulgaris* and *P. mirabilis* were susceptible to cefotaxime and amikacin. There was 100% resistant of *P*. *mirabilis* to cotrimoxazole and cefazolin while 50% and 100% of *P. vulgaris* isolates were resistant to cotrimoxazole and cefazolin respectively. All isolates (100%) of *C*. *freundii* were resistant to ampicillin and cefazolin while 33.3% (1/3) were sensitive to ciprofloxacin, cotrimoxazole, cefotaxime, gentamycin and ceftriaxone (Table 3).

**Table 3:** Antibiotic susceptibility test result of Gram negative bacteria isolated from pus specimens

Isolates	Antimicrobial agents									
	RXN	AMP	AK	CIP	COT	GEN	CTX	CTR	CZ	MRP
*E.. coli* (10)	S	0	Nt	6 (60)	4 (40)	8 (80)	5 (50)	3 (30)	2 (20)	Nt
	R	10 (100)	Nt	4 (40)	6 (60)	2 (20)	5 (50)	7 (70)	8 (80)	Nt
*P. aeruginosa* (5)	S	Nt	5 (100)	4 (80)	Nt	Nt	Nt	Nt	Nt	Nt
	R	Nt	0	1 (20)	Nt	Nt	Nt	Nt	Nt	Nt
*K. pneumoniae* (6)	S	Nt	4 (66.7)	4 (66.7)	3 (50)	4 (66.7)	Nt	3 (50)	Nt	5 (83.3)
	R	Nt	2 (33.3)	2 (33.3)	3 (50)	2 (33.3)	Nt	3 (50)	Nt	1 (16.7)
*P*. *vulgaris* (*n* = 2)	S	0	2 (100)	1 (50)	1 (50)	Nt	2 (100)	Nt	0	Nt
	R	2 (100)	0	1 (50)	1 (50)	Nt	0	Nt	2 (100)	Nt
*P*. *mirabilis* (*n* = 1)	S	1 (100)	1 (100)	1 (100)	0	Nt	1 (100)	Nt	0	Nt
	R	0	0	0	1 (100)	Nt	0	Nt	1 (100)	Nt
*C*. *freundii (3)*	S	0	Nt	1 (33.3)	1 (33.3)	1 (33.3)	1 (33.3)	1 (33.3)	0	Nt
	R	3 (100)	Nt	2 (66.7)	2 (66.7)	2 (66.7)	2 (66.7)	2 (66.7)	3 (100)	Nt
*Acinetobacter* spp. (*n* = 6)	S	2 (33.3)	4 (66.7)	4 (66.7)	3 (50)	4 (66.7)	3 (50)	Nt	3 (50)	Nt
	R	4 (66.7)	2 (33.3)	2 (33.3)	3 (50)	2 (33.3)	3 (50)	Nt	3 (50)	Nt
	Antimicrobial agents									
	RXN	AMP	AK	CIP	CAZ	TOB	IMP	PI	CZ	MRP
*P. aeruginosa* (5)	S	Nt	5 (100)	4 (80)	2 (40)	5 (100)	5 (100)	3 (60)	Nt	Nt
	R	Nt	0	1 (20)	3 (60)	0	0	2 (40)	Nt	Nt
Total (*n* = 38)	S	3 (13.6)	21 (84)	25 (65.7)	14 (42.4)	22 (73.3)	17 (63)	10 (41.7)	5 (22.7)	5 (83.3)
	R	19 (86.4)	4 (16)	13 (34.3)	19 (57.6)	8 (26.7)	10 (37)	14 (58.3)	17 (77.3)	1 (16.7)

*Nt* not tested, S Sensitive, *R* Resistant, *RXN* Reaction, *AMP* Ampicillin, *AK* Amikacin, *CIP* Ciprofloxacin, *COT* trimethoprim + sulfamethoxazole (cotrimoxazole), *GEN* Gentamicin, *CTX* Cefotaxime, *Caz* Ceftazidime, *TOB* Tobramycin, *IMP* Imipenem, *PI* Piperacillin, *CTR* Ceftriaxone, *CZ* Cefazolin, *MRP* Meropenem

#### Antibiogram result of gram positive *S*. *aureus*, CoNS, and *Enterococcus* species

Among total isolated *S*. *aureus*, 77.3% of *S*. *aureus* were susceptible to gentamycin, where 75.8% of the isolates were susceptible to cefotaxime. Similarly, 45.5% of *S*. *aureus* were susceptible to ciprofloxacin while 39.4% of *S. aureus* isolates were susceptible to cefoxitin. Eighty percent of *Enterococcus* spp. were sensitive to tetracycline. (Table 4). Among 66 *S*. *aureus* isolated from pus swab and aspirated pus, 40 (60.6%) isolates of *S*. *aureus* were MRSA.

**Table 4:** Antibiotic susceptibility test result of Gram positive bacteria isolated from pus specimens

Isolates	Antimicrobial agents								
	RXN	AMP	AK	CIP	COT	GEN	CTX	CX	TE
*S*. *aureus* (*n* = 66)	S	5 (7.6)	Nt	37 (56.1)	26 (39.4)	54 (81.8)	53 (80.3)	26 (39.4)	29 (43.9)
	R	61 (92.4)	Nt	29 (43.9)	40 (60.6)	12 (18.2)	13 (19.7)	40 (60.6)	37 (56.1)
CoNS (*n* = 9)	S	1 (11.1)	Nt	3 (33.3)	4 (44.4)	6 (66.7)	2 (22.2)	4 (44.4)	5 (55.6)
	R	8 (88.9)	Nt	6 (66.7)	5 (55.6)	3 (33.3)	7 (77.8)	5 (55.6)	4 (44.4)
*Enterococcus* spp. (*n* = 5)	S	3 (60)	2 (40)	3 (60)	3 (60)	3 (60)	3 (60)	Nt	4 (80)
	R	22 (40)	3 (60)	2 (40)	2 (40)	2 (40)	2 (40)	Nt	1 (20)
Total (*n* = 80)	S	9 (11.25)	2 (40)	43 (53.75)	33 (41.25)	63 (78.75)	58 (72.5)	30 (40)	38 (47.5)
	R	71 (88.75)	3 (60)	37 (46.25)	47 (58.75)	17 (21.25)	22 (27.5)	45 (60)	42 (52.5)

*Nt* not tested, *S* Sensitive, *R* Resistant, *RXN* Reaction, *AMP* Ampicillin, *AK* Amikacin, *CIP* Ciprofloxacin, *COT* trimethoprim + sulfamethoxazole (cotrimoxazole), *GEN* Gentamicin, *CTX* Cefotaxime, *CX* Cefoxitin, *TE* Tetracycline

#### ESBL producers among Enterobacteriaceae isolates

Among 10 isolates of *E*. *coli*, 2 (25%) were positive for ESBL and among 6 isolates of *K*. *pneumoniae*, 2 (40%) were positive for ESBL. Additionally, among 3 isolates of *C*. *freundii*, 1 (33.3%) was ESBL positive whereas *Proteus* spp. were negative for ESBL (Table 5).

**Table 5:** ESBL producers among Enterobacteriaceae

Bacterial isolates	Total	ESBL producer	
		No.	%
*E*. *coli*	10	2	25.0
*K*. *pneumoniae*	6	2	40.0
*P*. *vulgaris*	2	0	0
*P*. *mirabilis*	1	0	0
*C*. *freundii*	3	1	33.3

#### Antibiogram result of isolates

Eighty percent (80%) of *E. coli* and 68.2% of *S. aureus* were MDR (resistant to two or more than two antimicrobial classes) strains. Similarly, 80% of *P. aeruginosa* and 77.7% of CoNS were MDR strains. Additionally, 83.3% of *K*. *pneumoniae* isolates were resistant to at least two different classes of used antibiotics. In this study, 50% of *Proteus* spp. isolates were MDR **(**Table 6**)**.

**Table 6:** Antibiogram result of isolates

Isolated organisms	Antibiogram				Total MDR [N(%)]
	No. (%) of resistance				
	R2	R3	R4	R5	
Gram positive					
*S. aureus* (*n* = 66)	20 (30.3)	18 (27.3)	3 (4.5)	4 (6.1)	45 (68.2)
CoNS (*n* = 9)	4 (44.4)	1 (11.1)	2 (22.2)	0	7 (77.7)
*Enterococcus* spp. (*n* = 5)	3 (60)	0	1 (20)	0	4 (80)
Total (*n* = 80)	27 (33.75)	19 (23.75)	6 (7.5)	4 (5)	56 (70)
Gram negative					
*E. coli* (*n* = 10)	6 (60)	1 (10)	0	1 (10)	8 (80)
*P. aeruginosa* (*n* = 5)	2 (40)	1 (20)	1 (20)	0	4 (80)
*Acinetobacter* spp. (*n* = 6)	2 (33.3)	1 (16.7)	1 (16.7)	0	4 (66.7)
*C*. *freundii* (*n* = 3)	2 (66.7)	0	0	0	2 (66.7)
*K*. *pneumoniae* (*n* = 6)	2 (33.3)	1 (16.7)	0	2 (33.3)	5 (83.3)
*P*. *vulgaris* (*n* = 2)	1 (50)	0	0	0	1 (50)
*P*. *mirabilis* (*n* = 1)	1 (50)	0	0	0	1 (50)
Total (*n* = 33)	10 (30.3)	3 (9.1)	2 (6.1)	2 (6.1)	17 (51.5)

*R2-R5* number of antibiotics class where an isolate was resistant

## Discussion

Aerobic bacteria causing wound infections were isolated and identified from pus specimens by series of biochemical tests and their antimicrobial susceptibility patterns to commonly used antibiotics in study area were examined. Enterobacteriaceae isolates were further processed for confirmation of ESBL producer. In this study, 60.4% of culture positive specimens showed monomicrobial growth, 1.7% showed polymicrobial and 37.9% were negative for aerobic bacterial growth. This finding is consistent with previous studies conducted by Egbe et al. and Kumari et al. [19, 20]. Bhatta et al., [21] have reported 60% of bacterial wound infection from Nepal in 2008. Out of 182 non-repeated samples analyzed, 143 (78.6%) samples were from inpatients, where 98 (68%) were positive for aerobic bacterial growth. Our finding shows higher rate of wound infection in inpatients (68%) as compare to outpatients (39%) and the result was statistically significant (*p* < 0.05). Similar finding was reported by Stephen et al. [19]. Among 182 specimens collected, 156 (85.7%) were pus swabs with 64% (100/156) aerobic bacterial growth and 26 (14.3%) were aspirated pus where 13 (50%) were positive for aerobic bacterial growth. Shrestha et al., [21] have found the similar prevalence rate in Nepal before. Pus aspiration is generally taken as sample of choice from deep seated and closed wound infections [22, 23].

Eighty one (44.5%) pus specimens were collected from male patients, while 101 (55.5%) specimens were from female patients and the result was statistically insignificant (*p* > 0.05). In this study, female patients outnumbered the male patients [24] but other studies showed wound infection was higher in male as compared to female [25, 26]. In our study, lower number of male patients (44.5%) might be due to small sample size as compared to other studies. In this study, monomicrobial growth (97.3%) was higher than polymicrobial growth (2.7%) both in pus swab and aspirated pus. Multiple studies carried out in wound infections have shown higher rate of monomicrobial infection than polymicrobial infection [27]. Similarly a high rate (86–100%) of monomicrobial wound infection was reported from different states of India [28, 29].

Among different age groups, the prevalence of wound infections was highest among age group ≤10 years (82.1%) followed by age group 70–80 years (77.8%). This is in agreement with study carried by Lakhey et al. where higher prevalence of wound infection was reported among patients of age group 60–80 years [20]. Similarly, in a study done by Mohammedaman et al., [5] in South Ethiopia, 87.5% wound infection was in patients with age ≥ 60 years. Since old individuals and children have weak immunity, that might be the reason for them being more prone to wound infections. Ranjan et al. have reported more pathogenic strains from patients of age group 21–40 years in post-operative wound infections in India [30].

Among 116 bacterial isolates, 11 different species were identified. *S*. *aureus* (56.9%) was the most common isolate followed by *E*. *coli* (8.6%) and CoNS (7.8%). Other identified bacteria from pus specimens included *P. aeruginosa* (4.3%), *Acinetobacter* spp. (5.2%), *Enterococcus* spp. (4.3%), *C. freundii* (2.6%), *K. pneumoniae* (5.2%), *P*. *vulgaris* (1.6%), and *P. mirabilis* (0.9%). The predominance of *S*. *aureus* in wound infection is supported by different studies [21, 30]. As being a normal flora of human skin, it can get access into the wound easily. Kansakar et al., [32] have reported that 82.5% of bacterial growth in pus samples and 13 different bacterial species were isolated where *S*. *aureus* was predominant (57.7%) species followed by *E*. *coli* (11%) and CoNS (3%). According to Mumtaz et al., [33] *S*. *aureus* was the most common bacteria (49%) found in wound infections followed by *E*. *coli* (25.9%), *Klebsiella* spp. (9.5%), *P*. *aeruginosa* (8.6%), *Proteus s*pp. (4%) and *Acinetobacter* (2.7%) spp. *S*. *aureus* is the most common strain (25%) as a commensal organism of human skin and nasal passage. Hence, most frequent isolation of *S*. *aureus* from pus specimens might also be due to contamination of collected specimens with skin normal flora [31]. Contribution of multidrug resistant *Acinetobacter* spp. to nosocomial infections has increased over the past decade, and many outbreaks involving this bacterium have been reported worldwide [32].

Shrestha et al., [21] have found that 85% of *S*. *aureus* isolates were sensitive to ciprofloxacin, 83% and 82% were sensitive to cephalexin and cotrimoxazole respectively. In this study, 60.6% of Staphylococci isolates were resistant to cefoxitin. *S*. *aureus* which was resistant to cefoxitin antibiotic was reported as MRSA species. Rajbhandari et al., [36] have also reported 61.6% of MRSA prevalence in wound infection. The second common isolate of this study was *E*. *coli* where 80%, 60%, 50% and 40% of the isolates were susceptible to gentamycin, ciprofloxacin, cefotaxime and cotrimoxazole respectively. All the isolates of *E*. *coli* (100%) were resistant to ampicillin where 30% and 20% were resistant to ceftriaxone and cefazolin respectively. Similarly, 60% and 40% of *E. coli* isolates were susceptible to ciprofloxacin and cotrimoxazole respectively. This study showed low sensitivity rate as compared to other studies [33]. Hence, increased antimicrobial resistant rate of *E. coli* depicts its important role in nosocomial infections.

All the isolates of *P*. *aeruginosa* (100%) were sensitive to amikacin, tobramycin and imipenem while 80% and 60% were sensitive to ciprofloxacin and piperacillin respectively. Only 40% of the *P*. *aeruginosa* were susceptible to the antibiotic ceftazidime. In a study conducted by Shrestha et al., [21] 93% of isolates were sensitive to amikacin and 66.7% of isolates were sensitive to ciprofloxacin. Our finding in this context is similar with other results where *P. aeruginosa* isolated from pus samples has shown least resistance to ciprofloxacin (6.2–24%) [34]. More prevalence of antimicrobial resistant *P. aeruginosa* in wound infection is being a challenging issue especially in resource limited countries [26].

*K*. *pneumoniae* was most sensitive to meropenem (83.3%) and 66.7% of *K*. *pneumoniae* isolates were equally resistant to gentamycin, ciprofloxacin, and amikacin where 50% of isolated *K*. *pneumoniae* were resistant to cotrimoxazole and ceftriaxone. In a study reported by Mohammedaman et al., [5] 35.7% of *K*. *pneumoniae* were resistant to ciprofloxacin and doxycycline. Furthermore, Rajput et al., [24] had reported that 45.5% and 80% of *K*. *pneumoniae* strains were resistant to ciprofloxacin and cotrimoxazole respectively. All isolates (100%) of *P*. *vulgaris* were susceptible to amikacin, and cefotaxime but 100% of *P*. *vulgaris* isolates were resistant to ampicillin and cefazolin while 50% of isolated *P*. *vulgaris* were resistant to ciprofloxacin and cotrimoxazole. All isolates (100%) of *P*. *mirabilis* were sensitive to ciprofloxacin, amikacin and cefotaxime whereas 100% were resistant to ampicillin, cotrimoxazole and cefazolin. This result is comparable with study carried by Bhatta et al. [20].

Among Enterobacteriaceae isolates, 25% of *E*. *coli*, 40% of *K*. *pneumoniae* and 33.3% of *C*. *freundii* were ESBL producer. But none of the *Proteus* species were ESBL producer. Chander et al., [35] have reported 13.51% and 16.55% of *E*. *coli* and *K*. *pneumoniae* as ESBL producer respectively. The prevalence rate may vary based on sample collection method, site of sample collection, microbial detection technique, antimicrobial agents used, and geographical location. In this study, 68.2% of *S*. *aureus* and 80% of *E. coli* isolates were MDR strains. The highest rate (83.3%) of MDR was observed in *K*. *pneumoniae*. This finding is in agreement with the study conducted in South-West Ethiopia by Mohammedaman et al. [5]. Most of the Gram negative isolates were resistant to ampicillin (86.4%) and cefazolin (77.3%) while 88.6% and 60% of Gram positive bacteria were resistant to ampicillin and amikacin respectively. In Nepal, oral administration of antibiotics is common practice which may reduce absorption of antibiotics by blood stream. Long term use of antibiotics via oral route could contribute to bacteria developing resistance.

Wound infection is a burning public health issue especially in developing countries. Severe wound infection can cause great loss including higher rate of morbidity and mortality; longer hospital stays, delay in wound healing, increase economic burden and increase discomfort which in turn increases disease burden significantly. Wound infection is being a common nosocomial infections which accounts for 0–80% of patient’s mortality [35, 36].

Modernization in control and prevention of infections has not completely controlled wound infection due to increasing problem of antimicrobial resistance [37]. As compared to previous studies, antimicrobial resistance pattern is increasing at high rate. Multiple factors may contribute to rapid development of antimicrobial resistance by pathogens including misuse, overuse, and underuse of antimicrobials by both clinicians and patients. In Nepal, people purchase antimicrobials without physician’s prescription, which is a common practice. This leads to misuse of antimicrobials that contributes to the emergence and spread of antimicrobial resistant strain. MRSA and ESBL producing bacteria are creating a serious problem in wound treatment in different parts of the country.

## Conclusion

In this study, the most common isolate was *S. aureus* in pus specimens. Among *S. aureus* isolates*,* 60.6% were MRSA strains, whereas 40% of *K. pneumoniae* and 33.3% *C. freundii* were ESBL producer followed by *E. coli* (25%). Eighty percent (80%) of *E. coli*, *P. aeruginosa*, and 68.2% of *S. aureus* were MDR strains. This study emphasizes the importance of strict nosocomial infection control strategies and careful prescription of antimicrobials should be implemented by the health care centres. It should be mandatory to screen out ESBL, MRSA, and MDR pathogens and regular monitoring of their antimicrobial susceptibility pattern for prevention and control of wound infections. Early reporting of drug resistant pathogens and evidence-based treatment algorithm can control the wound infections. Research on AMR is in its infancy stage in Nepal, but it is paramount to establish surveillance programs to reduce burden of wound infections.

## Additional file

Additional file 1: Photograph file. (DOCX 777 kb)

## Abbreviations

AMR: Antimicrobial resistance
AST: Antibiotic susceptibility test
ATCC: American type culture collection
CDC: Centers for disease control and prevention
CLSI: Clinical laboratory standard institute
CoNS: Coagulase negative Staphylococci
DDST: Double disc synergy test
ENT: Eye-Nose-Throat
ESBL: Extended spectrum β-lactamase
FNAC: Fine needle aspiration cytology
MDR: Multi-drug resistant
MHA: Mueller Hinton agar
MRSA: Methicillin resistant *Staphylococcus aureus*
SPSS: Statistical package for the social sciences
WHO: World health organization
ZOI: Zone of inhibition
μg: Micro gram
