# Supplementary material for: Prevalence of methicillin resistant Staphylococcus aureus, multidrug resistant and extended spectrum β-lactamase producing gram negative bacilli causing wound infections at a tertiary care hospital of Nepal
Source: Antimicrob Resist Infect Control. 2018 Oct 8;7:121. doi: 10.1186/s13756-018-0408-z (PMC6174564; doi:10.1186/s13756-018-0408-z)
Supplement: Supplementary file 1 — Photograph file. (DOCX 777 kb) [file 13756_2018_408_MOESM1_ESM.docx]

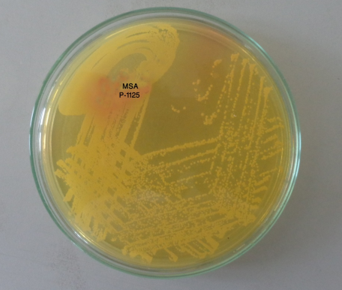


**Photograph 1:** Golden yellow colonies of *Staphylococcus aureus* on MSA medium (**Sample code: p-146**).


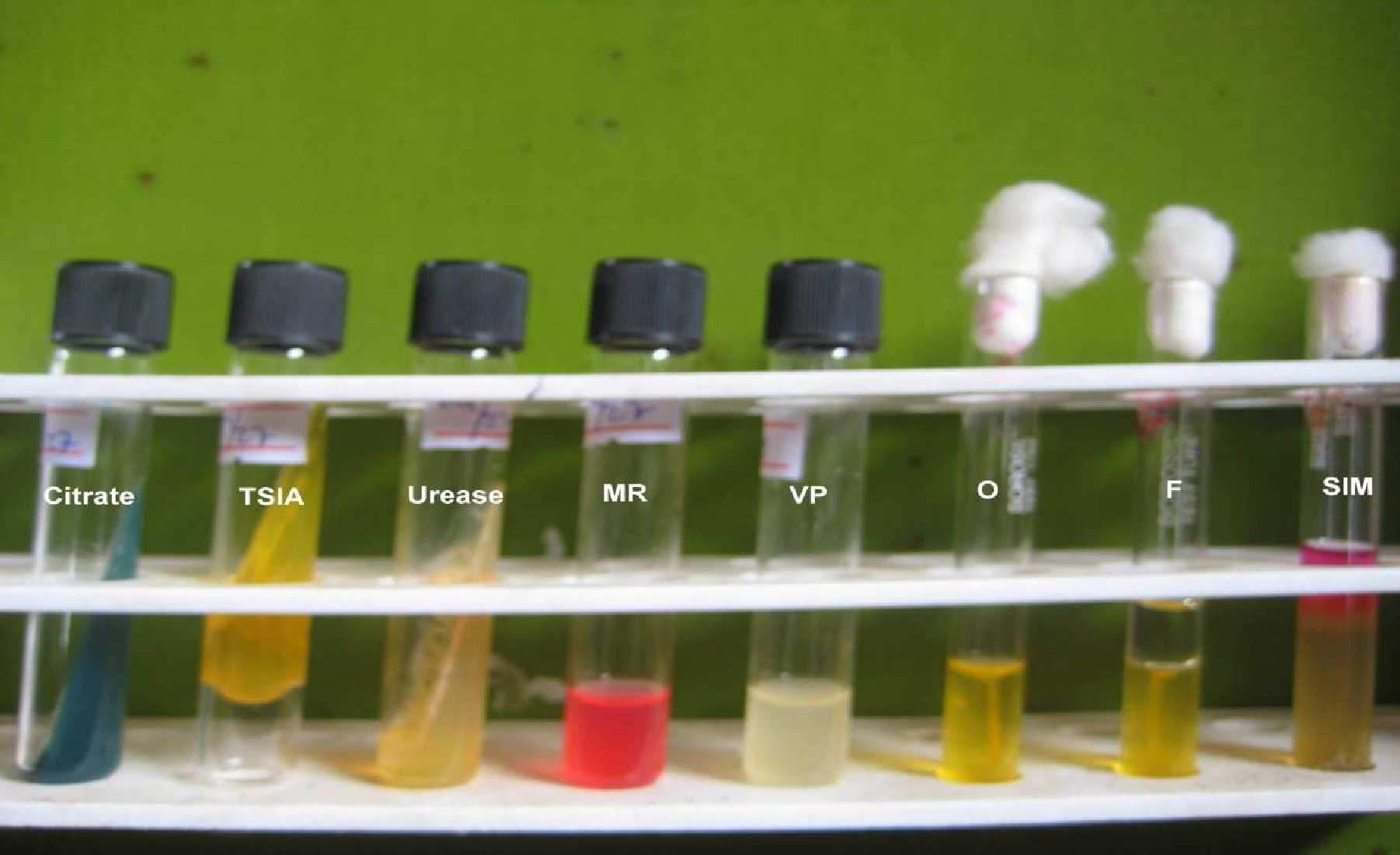


**Photograph 2:** Biochemical tests of *Escherichia coli* (Citrate= -ve, TSIA= +ve, Urease= -ve, MR= +ve, VP= -ve, OF= fermentative, H_2_S= -ve, Indole= +ve, motile) (**Sample code: p-76**).


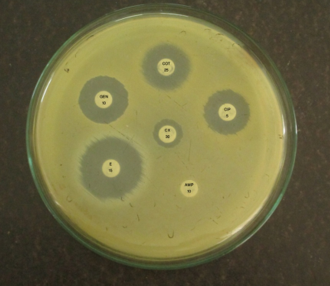


**Photograph 3:** Antibiotic susceptibility pattern of *Staphylococcus aureus* where cefoxitin (CX) = resistant, ampicillin (AMP) = resistant, erythromycin (E) = sensitive, gentamicin (G) = Sensitive, ciprofloxacin (CIP) = sensitive and co-trimoxazole (COT) = sensitive (**Sample code: p-43**).


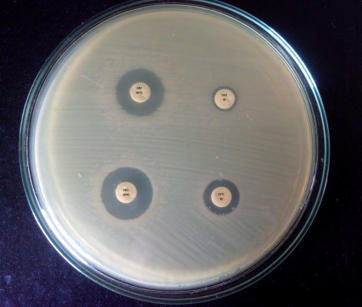


**Photograph 4:** Positive ESBL confirmation test on Mueller- Hinton Agar (CAZ 30 and CAC 30/10 and CTX 30 and CEC 30/10) (Difference in zone of inhibition of CAC and CAZ >5mm) (**Sample code: p-167**).
